# The Role of Programmed Necrosis in Colorectal Cancer

**DOI:** 10.3390/cancers14174295

**Published:** 2022-09-01

**Authors:** Yu-Qiang Yu, Reyes Gamez-Belmonte, Jay V. Patankar, Eva Liebing, Christoph Becker

**Affiliations:** 1 Department of Medicine 1, Friedrich-Alexander-Universität Erlangen-Nürnberg (FAU), 91054 Erlangen, Germany; 2 Deutsches Zentrum Immuntherapie (DZI), 91054 Erlangen, Germany; 3 Department of Immunology, Duke University School of Medicine, Durham, NC 27710, USA

**Keywords:** necroptosis, pyroptosis, ferroptosis, colorectal cancer

## Abstract

**Simple Summary:**

Necrosis is a type of cell death characterized by plasma membrane rupture and the induction of inflammation. This review focuses on colorectal cancer and outlines the role of programmed necrosis in tumor development. Potential strategies for anti-tumor treatment via targeting programmed necrosis are also discussed.

**Abstract:**

For quite a long time, necrosis was considered a chaotic and unorganized form of cell death. However, studies conducted during the past few decades unveiled multiple types of programmed necrosis, such as necroptosis, pyroptosis and ferroptosis. These types of programmed necrosis have been shown to play crucial roles in mediating pathological processes, including tumorigenesis. Almost all key mediators, such as RIPK3 and MLKL in necroptosis, GSDMD and caspase 1/11 in pyroptosis and GPX4 in ferroptosis, are highly expressed in intestinal epithelial cells (IECs). An aberrant increase or decrease in programmed necrosis in IECs has been connected to intestinal disorders. Here, we review the pathways of programmed necrosis and the specific consequences of regulated necrosis in colorectal cancer (CRC) development. Translational aspects of programmed necrosis induction as a novel therapeutic alternative against CRC are also discussed.

## 1. Introduction

The human gut is a complex organ with a surface area of more than 30 m^2^ that is involved in multiple critical processes, including nutrient absorption and digestion, waste excretion and barrier regulation [1]. It deals with tremendous environmental challenges, such as microbial antigens and food allergens. These challenges alone or together with genetic predispositions may affect intestinal homeostasis, which, in turn, leads to intestinal disorders. Colorectal cancer (CRC) is a major health challenge and has been identified as the third most frequent type of cancer [2]. The progressive transformation of intestinal epithelial cells (IECs) into cancerous cells results in colorectal tumorigenesis [3]. Both IEC and colorectal tumor cells have been shown to be sensitive to multiple types of cell death [4,5].

Although remarkable progress in anti-tumor treatment has been achieved during the last few years, improving the overall prognosis remains a big challenge. In this sense, strategies developed to induce cell death in tumor cells have shown great potential in anti-tumor treatments. Apoptosis was used mostly synonymously with regulated cell death for quite a long time and several apoptosis inducers have been used for anti-tumor treatment [6]. However, caspase 8, a key molecule in mediating apoptosis, is frequently inactivated in human cancers [7,8]. The inactivation of caspase 8 in tumor cells blocked apoptosis and sensitized cells to necroptosis, a form of programmed necrosis [9]. Besides necroptosis, our knowledge about other types of programmed necrosis, such as caspase-dependent pyroptosis and caspase-independent ferroptosis, has also dramatically expanded. All three programmed necrosis forms can be therapeutically targeted [10,11,12]. Accumulating data suggest that programmed necrosis regulates CRC development [13,14,15]. In this review, we aim to summarize the existing data on the role of programmed necrosis in colorectal cancer and highlight the possibility of therapeutic interventions.

## 2. An Overview of Programmed Necrosis

Based on the morphological characteristics, most cell death processes can be identified as apoptosis or necrosis. Apoptosis is characterized by the shrinkage of cells and fragmentation into membrane-bound apoptotic bodies, while necrosis is described as a process involving membranous swelling of the organelles and DNA degradation [16]. Apoptosis was considered as the only type of programmed cell death before programmed necrosis was identified. Programmed necrosis is defined as a genetically controlled cell death mode with necrotic morphological features [17]. Necroptosis, pyroptosis and ferroptosis are three well-known forms of programmed necrosis.

**Figure 1:**
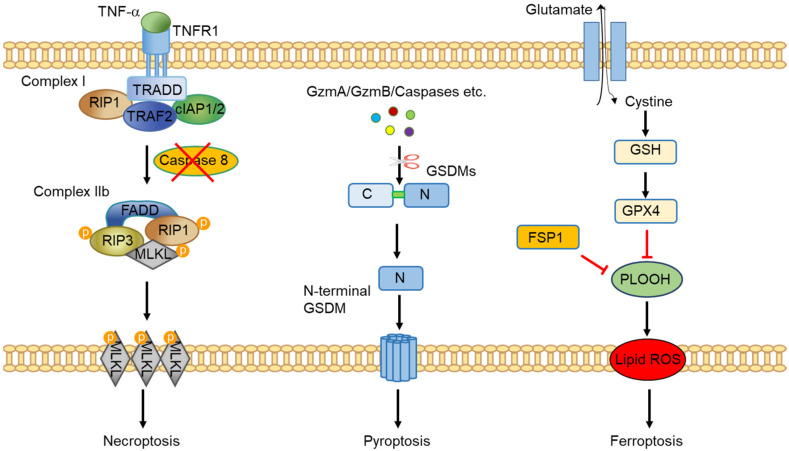
Key programmed necrosis pathways. The classical necroptotic pathway is activated by TNF stimulation. TNF binds to TNFR1 and leads to the formation of complex I. In the absence of caspase 8 activity, RIP1 interacts with RIPK3, FADD and MLKL to form complex IIb, which mediates necroptosis. Pyroptosis has been considered as gasdermin-mediated necrosis. Endogenous caspases and lymphocyte-derived Granzyme A/Granzyme B are able to cleave and activate individual gasdermins (GSDM) which further triggers pyroptosis. Ferroptosis is a failsafe rather than a typical cell death pathway. GPX4 and FSP1 prevent lipid peroxidation which is key to drive ferroptosis.

***Necroptosis*** was the first discovered programmed necrosis (Figure 1). It is induced upon caspase 8 inhibition/deficiency and TNFα stimulation and induces cell death via the necroptotic pathway [18]. Aside from TNFα, a number of other stimuli have been shown to be able to induce necroptosis via targeting their specific receptors. These receptors include other members of the Tumor Necrosis Factor Receptor (TNFR) superfamily, Pattern Recognition Receptors (PRRs), T Cell Receptors (TCRs) and Z-DNA-binding protein 1 (ZBP1) [19]. Of the different stimuli, TNF-induced necroptosis is currently the best-characterized necroptotic pathway. The binding of TNF to the TNFR1 induces a conformational change in TNFR1 receptor trimers, leading to the formation of complex I, a multi-protein complex comprising receptor-interacting serine/threonine-protein kinase 1 (RIPK1), TNFR-associated death domain (TRADD), TNF receptor-associated factor 2 (TRAF2), cellular inhibitor of apoptosis protein 1/2 (cIAP1/2) and others [20]. Within complex I, RIPK1 plays a crucial role in regulating cell fate. Upon polyubiquitination by cIAP1/2, RIPK1 and other components reinforce complex I and drive nuclear factor kappa B (NF-κB) activation, which promotes cell survival [21]. On the other hand, RIPK1 can be deubiquitinated by cylindromatosis (CYLD), leading to the suppression of the NF-κB pathway and instead, activation of cell death pathways. In that situation, a protein complex known as complex II forms. It still includes RIPK1, TRADD, TRAF2 and CIAP1/2, but now also FADD, caspase-8 and RIPK3 can be part of the complex with the potential to drive apoptosis and necroptosis. In the presence of caspase 8, RIPK1 and RIPK3 are cleaved and cells undergo apoptosis. However, upon pharmacological inhibition or genetic deficiency of caspase 8, RIPK1 and RIPK3 auto- and transphosphorylate each other, leading to the formation of a microfilament-like complex called the necrosome that directs cells to necroptosis [21]. Mixed Lineage Kinase Domain-like (MLKL), an effector protein downstream of RIPK1 and RIPK3, executes necroptosis when phosphorylated via plasma membrane translocation. In addition to the canonical necroptosis pathway, studies also identified that programmed necrosis, under certain conditions, can still occur when one of these mediators is missing. For example, MLKL mediates programmed necrosis in hepatocytes independently of RIPK3 [22]. Necroptosis seems to be a complex cell death process. Defining its exact role in physiological and pathophysiological processes and in relation to apoptosis requires studies in different organs and model systems.

***Pyroptosis*** is a type of gasdermin-mediated necrosis (Figure 1). Six proteins have been included in the gasdermin protein family so far: GSDMA, GSDMB, GSDMC, GSDMD, GSDME (also known as DFNA5) and DFNB59 (also known as pejvakin) [23]. Members of this family contain a C-terminal and an N-terminal domain. The N-terminal domain harbors activity in executing pyroptosis while the C-terminal domain inhibits the activity [24]. In order to mediate pyroptosis, the N-terminal domain has to be intact and liberated from the C-terminal domain by cleavage. Of this group of proteins, GSDMD is the most extensively studied [25]. The linker region between the N- and C-terminal domains of GSDMD is generally cleaved by inflammatory caspases, such as caspase 1, caspase 8 and caspase 4/5/11 [26]. In addition to these caspases, cathepsin G and neutrophil-derived elastase are also able to release the N-terminal domain of GSDMD [27,28]. Upon release, the N-terminal domain translocates to the membrane where it assembles into a pore structure via a Ragulator-Rag-mTORC1-dependent pathway [29]. As a result, small molecules, such as IL-1β, are released from the cells via these pore structures. Subsequently, the cell-surface protein Nerve Injury-induced Protein 1 (NINJ1) mediates plasma membrane rupture and results in the release of lactate dehydrogenase (LDH) and other large damage-associated molecular patterns [30]. Interestingly, unlike the caspases mentioned above, which activate GSDMD, caspase 3 has been shown to cleave the N-terminal region of GSDMD, thereby inhibiting its function in pyroptosis induction [31]. In addition to GSDMD, the N-terminal domains released from granzyme A-cleaved GSDMB, caspase 8-cleaved GSDMC or granzyme B/caspase 3-cleaved GSDME have also been shown to mediate pyroptosis [25,32,33,34,35,36]. GSDMA has recently been reported to be cleaved by a protease virulence factor, SpeB, released by human pathogen group A Streptococcus [37].

***Ferroptosis*** is another recently identified form of programmed necrosis. Ferroptosis is mediated by iron-dependent accumulation of lipid peroxidation (Figure 1) and dysfunction of molecules that prevent lipid peroxidation results in the execution of ferroptotic cell death [38]. Glutathione peroxidase 4 (GPX4), a phospholipid hydroperoxidase that converts glutathione into oxidized glutathione to reduce cytotoxic lipid peroxides, is the most-studied molecule in ferroptosis [39]. Defects in glutathione synthesis or deficiency/inhibition of GPX4 result in phospholipid hydroperoxide (PLOOH) accumulation in cells and subsequently, leads to the induction of ferroptosis. RSL3 is frequently used as a GPX4 inhibitor to induce ferroptosis while erastin induces ferroptosis by blocking glutathione synthesis via targeting cysteine import. Together with genetic tools, these two pharmacological inhibitors are indispensable for ferroptosis research. More recently, GPX4-independent pathways of ferroptosis suppression have been identified. Ferroptosis suppressor protein 1 (FSP1), also known as apoptosis-inducing factor mitochondria associated 2 (AIFM2), has been shown to be recruited to the plasma membrane to prevent ferroptosis [40,41]. In addition, GTP cyclohydrolase-1 (GCH1) and prominin 2 were identified as negative regulators of ferroptosis [42,43]. It is now widely accepted that ferroptosis is a type of cell death involved in oxidative and metabolic stress. Cells, which show active metabolism and ROS overload, such as cancer cells, are potentially more sensitive to ferroptosis. Mutations of several oncogenes, including *RAS* and *TP53,* have been associated with ferroptosis inhibition [44]. Furthermore, epithelial-to-mesenchymal transition (EMT), a process believed to generate cancer stem cells and metastasis, sensitizes tumor cells to ferroptosis [45,46]. Thus, targeting ferroptosis may provide new strategies for cancer therapy.

## 3. Programmed Necrosis in Colorectal Tumor Cells

During the development of CRC, stress, including metabolic effects from the tumor microenvironment, triggers the activation of cell death pathways. Programmed necrosis is one of the major types of cell death in tumor cells [47]. Its induction has been shown to have dual effects: promoting or reducing tumor growth in different types of cancers. The induction of programed necrosis kills tumor cells and, therefore, inhibits tumor development. On the other hand, programmed necrosis, as lytic cell death, also releases intracellular contents into the extracellular milieu, which, in turn, triggers inflammatory responses and may promote tumor development. Here, we discuss the current knowledge of the roles of necroptosis, pyroptosis and ferroptosis in CRC development.

***Necroptosis*** in tumor cells appears to be a favorable factor for colorectal tumor clearance, as downregulation of several of the key molecules in necroptotic signaling, such as RIPK3 and MLKL, are related to poor prognosis in CRC [48,49,50]. The low expression of RIPK3 in human colorectal tumors was found to be associated with poor disease-free survival and overall survival. Functional roles of RIPK3 in CRC were confirmed in inflammation-associated and sporadic murine colorectal tumor models, as RIPK3 deficiency significantly aggravated tumor burden in both models [51,52]. In contrast to these studies, a recently published study reported that RIPK3 deficiency or MLKL deficiency had no impact on inflammation-associated or sporadic colorectal tumor development in mice [53], suggesting that necroptosis is rather irrelevant for CRC development. Further studies will be required to better understand these conflicting observations. However, different mouse backgrounds or microbiota might be underlying these differences, as genetic effects and gut microbiota have been identified as key players in regulating CRC development [54,55]. Of note, most of the current studies target MLKL or RIPK3 as a tool to study the consequence of blocking necroptosis in CRC development. However, accumulating data show necroptosis-independent functions of RIPK3 and MLKL [56,57]. More specific strategies for targeting necroptosis without affecting other pathways will be very helpful to dissect these functions. 

***Pyroptosis*** is characterized as the consequence of gasdermin activation. The role of pyroptosis in infectious diseases has been well established, while only a few studies have focused on tumor development and the exact roles of pyroptosis on CRC are largely unknown. Multiple strategies have been developed to induce pyroptosis in cultured tumor cells via targeting different gasdermins. Lobaplatin, an anti-tumor drug, has been shown to induce GSDME-dependent pyroptosis [58]. In a co-culture system, cytotoxic lymphocytes induced pyroptosis in tumor cells and the killing effects resulted from the cleavage of GSDMB by lymphocyte-derived granzyme A [32]. Interestingly, the robust pyroptosis induction observed in vitro seems not to directly affect tumor development in vivo, as GSDME deficiency or GSDMB overexpression failed to alter tumor growth [32,58]. One possible explanation for this discrepancy is the pro-inflammatory response induced by pyroptosis in vivo, which might promote tumor cell proliferation and compensate for the tumor cell loss associated with cell death. Of note, it has also been shown that GSDMD deficiency switches cells from pyroptosis to apoptosis and GSDME sufficiency switches cells from TNF-induced apoptosis to pyroptosis [35,59]. The possibility of switching types of cell death indicates that pyroptosis may serve as an alternative cell death under certain conditions. Thus, knocking out a particular gasdermin only blocks pyroptosis but other types of cell death may fill the gap, so the overall cell death remains constant with or without the gasdermin in vivo. Furthermore, pyroptosis-induced anti-tumor immunity should also be considered as GSDMB has been shown to dramatically improve anti-tumor effects of anti PD-1 therapy [32]. Interestingly, the effects of pyroptosis can also vary, depending on the models used. For example, GSDME-mediated pyroptosis has been shown to promote the development of colitis-associated colorectal cancer [14], but not in a xenograft model [58]. Little is known about the role of GSDMC in CRC, but the sparse evidence points to a functional role of GSDMC-induced pyroptosis in tumorigenesis. GSDMC expression is notably upregulated in experimental CRC as well as in CRC patients [60]. In 2016, it was shown that silencing GSDMC in CRC lines promotes tumor growth in vivo and in vitro, supporting the anti-tumor role of pyroptosis in CRC and uncovering GSDMC as a new oncogene [60]. Furthermore, a recent publication unveiled that in an acidic environment, tumor cells are sensitized to α-ketoglutarate (αKG)-induced pyroptosis [33]. In short, αKG boosts ROS level and caspase 8 activation, which, in turn, cleaves GSDMC. This opens the way of using αKG as a new strategy in CRC treatment through the induction of GSDMC-mediated pyroptosis [33]. 

***Ferroptosis*** was discovered in 2012 and, since then, the induction of ferroptosis by specific molecules has been shown to inhibit tumor growth in multiple cancer types, including CRC [61]. RSL3-targeted GPX4 inactivation is a classical method to induce ferroptosis and it has been shown to decrease CRC development in a xenograft mouse model [62]. Some chemotherapeutic drugs, such as cisplatin, have been shown to induce ferroptosis [63]. Cisplatin treatment depletes intracellular GSH and results in GPX4 inhibition-induced ferroptosis [64]. Moreover, the combination of cisplatin and classical ferroptosis inducers, such as erastin and RSL3, significantly enhance the anticancer effects of cisplatin [64,65,66]. In addition, targeting ferroptosis has been shown to overcome conventional CRC drug resistance [67,68]. Recent studies showed that cell density affects ferroptosis sensitivity via E-cadherin and the Hippo pathway [46,69]. As decreased E-cadherin and the Hippo pathway activity in tumor cells have been shown to associate with EMT and tumor metastasis [70], ferroptosis may also regulate tumor metastasis. Indeed, GPX4 inhibition has been shown to sensitize melanoma and breast tumor cells to ferroptosis and reduce metastatic capability [71,72]. This finding is especially interesting given that tumor metastasis is the leading cause of death in patients with cancers [73]. To the best of our knowledge, there is currently no in vivo evidence available regarding the role of ferroptosis in CRC metastasis. However, it is likely that metastatic CRC cells may also be sensitive to ferroptosis. Further studies are needed to prove or disprove this assertion.

## 4. Crosstalk between Programmed Necrosis and Anti-Tumor Immunity

**Figure 2:**
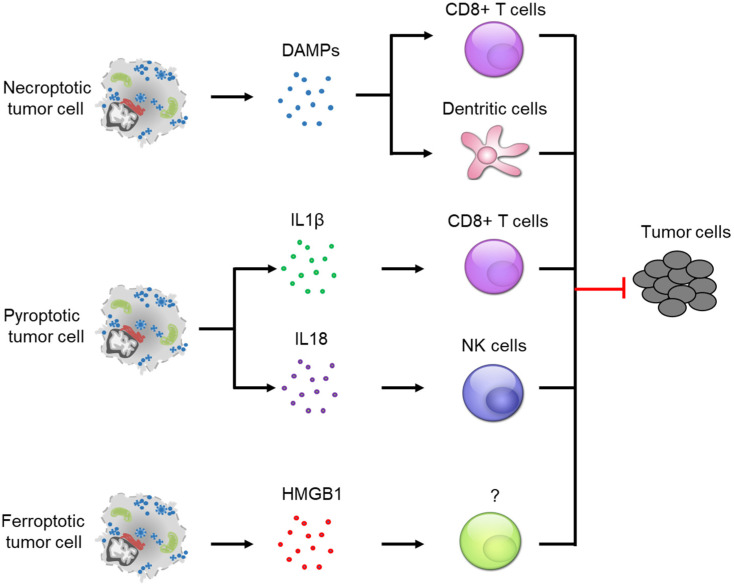
Programmed necrosis-induced anti-tumor immunity. The induction of necroptosis in tumor cells releases DAMPs and triggers CD8+ T cell- and dendritic cell-mediated tumor suppression. IL1β and IL18 derived from pyroptotic tumor cells can also trigger the CD8+ T cell and NK cell-mediated anti-tumor immunity, respectively. Ferroptosis induces anti-tumor immunity via the release of HMGB1.

Programmed necrosis has been considered as a highly inflammatory variant of cell death [74]. It involves the release of intracellular molecules called Danger-associated Molecular Patterns (DAMPs). The DAMPs seem necessary for the recruitment and maturation of immune cells in the tumor microenvironment. Recently, several types of programmed necrosis, including necroptosis, pyroptosis and ferroptosis, were reported as immunogenic cell death (ICD), associated with anti-tumor immunity [5] (Figure 2).

***Necroptosis*** was firstly confirmed as an ICD in 2016 and has been shown to be particularly involved in antigen presentation and cross-priming of CD8+ T cells [75,76,77]. Necroptotic tumor cells release massive DAMPs to trigger CD8+ T cell-mediated cytotoxic effects. The presence of necroptotic tumor cells also promotes proliferation of CD8+ T cells and maturation of bone-marrow-derived dendritic cells (DC) [77]. In support of this role, a recent study revealed the requirement of BATF3+ cDC1 cells and CD8+ leukocytes in necroptosis-induced anti-tumor immunity [78]. Using necroptotic tumor cells as a prophylactic tumor vaccination, in vivo studies showed very promising anti-tumor effects [76,77,78]. In addition to that, necroptotic fibroblasts can also be used for prophylactic tumor vaccination. Similar to the anti-tumor effects obtained from the experiments performed with necroptotic tumor cells, vaccination with necroptotic fibroblasts also dramatically decreases tumor burden [78]. Both vaccination strategies that use necroptotic tumor cells or fibroblasts induce anti-tumor immunity via CD8+ T cells [5]. However, different mechanisms were identified. Necroptotic tumor cells induce CD8+ T cell activation via a DAMP-dependent pathway while necroptotic fibroblasts induce DAMP-independent but NF-kB-dependent anti-tumor immunity [77,78]. Surprisingly, unlike the strong anti-tumor immunity observed by these two vaccination strategies, which conduct cell implantation, a genetically modified murine model showed a complete opposite result in colon tumor development. Defective necroptosis, due to RIPK3 depletion, increases anti-tumor immunity, as evidenced by an increase in tumor infiltrating CD8+ T cells as well as a reduction in myeloid-derived suppressor cells (MDSCs) and tumor-associated macrophages (TAMs) [79]. What is more, TAMs also exhibited a shift from tumor-promoting type 2 macrophages (M2) to tumor inhibiting type 1 macrophages (M1). A potential explanation for the observed discrepancy may be the necroptosis-independent roles of RIPK3, as RIPK3 has been shown to regulate intestinal cell proliferation [80]. It is possible that RIPK3 regulates anti-tumor immunity via a necroptosis-independent pathway. Further studies need be conducted to address the anti-tumor immunity in multiple in vivo models with better strategies to silence or activate necroptosis.

***Pyroptosis*** was identified as an inflammatory form of lytic cell death, which is mediated by gasdermin proteins. GSDMD is the best-studied gasdermin protein and the pathways leading to GSDMD-mediated pyroptosis involve the formation of inflammasomes. Inflammasomes are large multiprotein complexes that recruit executioner caspases, such as caspase 1 or caspase 4/5/11 [81]. These caspases are known for cleaving GSDMD upon pyroptosis induction. Therefore, pyroptosis is tightly connected to inflammasomes, especially the NLRP3 inflammasome [82]. NLRP3 mediates anti-tumor immunity in CRC via two key cytokines, IL18 and IL1β. The formation of NLRP3 has been shown to induce IL18 expression and NLRP3 deficiency decreased its expression [82]. NLRP3 deficiency increased colorectal tumor burden and liver metastasis in the AOM-DSS model via an IL18-dependent pathway. Additionally, IL18 administration significantly reduced the effects induced by NLRP3 deficiency [83,84]. NLRP3-mediated IL18 release has been shown to activate NK cells, which, in turn, inhibit tumorigenesis [85]. In transplantation tumor models, IL1β has been shown to be required for cross-priming CD8+ T cells [86]. Further studies also showed that exogenous IL1β delivery enhances antigen-dependent CD8+ T cell immunity [87,88]. In addition, IL1β administration also increased CD8+ T cell numbers in vivo via promoting cell trafficking and survival [89]. Given the crucial role of CD8+ T cells and NK cells in anti-tumor immunity, it is not surprising to find that pyroptosis induction decreased tumor growth [90]. Interestingly, studies have shown that pyroptosis can also be induced via inflammasome-independent pathways [32,58], raising the question whether pyroptosis induces anti-tumor immunity independent of inflammasome formation. Multiple immune cells have been shown to be necessary for triggering pyroptosis in tumor cells. For example, cytotoxic T lymphocytes and NK cells express and release granzyme A into targeted tumor cells to cleave and activate GSDMB, leading to tumor cell pyroptosis [32]. Both cell types can also release granzyme B to activate GSDME-mediated pyroptosis in tumor cells [36]. The studies mentioned above also showed that the combination of pyroptosis induction with immunotherapy significantly decreased tumor size, further indicating a strong connection between anti-tumor immunity and inflammasome-independent pyroptosis. 

***Ferroptosis*** is distinct from the other two forms of programmed necrosis, which are frequently induced during immune responses. It remains largely unknown whether ferroptosis induction affects anti-tumor immunity [5]. Tumor cells undergoing ferroptosis are known to release DAMPs, such as HMGB1, which is a key protein for inducing immune responses [91]. It is highly likely that ferroptosis is able to trigger immune response and that the immune system, in turn, affects the ferroptotic process. However, limited functional evidence is available at present, though we do know that CD8+ T cells sensitize tumor cells to ferroptosis via IFNγ-STAT1-mediated SLC7A11 down-regulation [92]. Another study provided indirect evidence, as immune-cell-derived IL4 and IL13 suppressed GPX4 expression while it increased ALOX15 expression in several cell types [93]. Since down-regulated GPX4 or up-regulated ALOX15 has been connected to ferroptosis induction [94], it is possible that immune-cell-derived IL4/IL13 expression sensitizes tumor cells to ferroptosis. On the other hand, ferroptosis induction in vivo has been shown to promote the recruitment of immune cells, especially neutrophils [19,95,96]. The mechanism behind neutrophil recruitment remains unknown. Further studies are needed in order to identify the role of ferroptosis in CRC.

## 5. Targeting Programmed Necrosis in Colorectal Cancer

CRC is a malignant tumor with a high mortality rate worldwide and, therefore, multiple treatments, including surgery, radiotherapy, chemotherapy, immunotherapy and biologics, are widely used to improve overall CRC survival [97]. Apoptosis was the first identified programmed cell death mode induced in tumors, following some of these treatments and since then, several treatments have been developed to induce apoptosis in tumor cells. Surprisingly, more and more patients have been found to be resistant to these treatments as tumor cells develop apoptosis resistance over time [98,99]. Therefore, targeting different types of programmed necrosis may provide a promising strategy for cancer therapy and drugs/treatments have been summarized elsewhere [5]. Radiotherapy, a widely used treatment in cancer therapy, was found to induce necroptosis in CRC cells [100]. Small-molecular-weight proteins, such as apoptin, have been shown to induce pyroptosis in cancer cells via the GSDME-dependent pathway [101]. In addition to the classical ferroptosis inducer RSL3, chemotherapy drugs, such as cisplatin or nanoelicitor, were found to induce ferroptosis in tumor cells [64,102,103]. Interestingly, additional therapeutic benefit has been achieved in tumor treatment when cell death induction was combined with immune checkpoint inhibition [5,78]. This effect may be partly due to a direct involvement of immune cells in programmed necrosis. Take an example of GSDMB-induced pyroptosis, its induction requires cytotoxic lymphocyte-released granzyme A [32]. In this case, T cell activation is crucial for pyroptosis induction and tumor growth inhibition. Meanwhile, induction of necroptosis and pyroptosis is associated with greater CD8+ T cell infiltration, as discussed above. The enrichment of CD8+ T cells facilitates immunotherapy [104]. Therefore, cell death induction and immunotherapy can have synergistic effects in anticancer therapy. Clinical trials combining immune checkpoint inhibitors with chemotherapy/radiotherapy have been recently reviewed elsewhere [5].

Despite the promising anti-tumor effects achieved by targeting programmed necrosis, concerns remain about these treatment strategies. The major concern is the inflammatory response induced by programmed necrosis [105]. Cells undergoing necroptosis, pyroptosis or ferroptosis release massive amounts of intracellular contents, including DAMPs, into the extracellular milieu, which trigger pro-inflammatory responses. The response may promote tumor cell proliferation or induce cytokine expression, which can be harmful for patients.

## 6. Conclusions

Our understanding about the ways of cells undergoing programed cell death has been accelerating over the past few decades. Several new types of programed necrosis, such as necroptosis, ferroptosis and pyroptosis, have been identified and more detailed knowledge of these pathways has been revealed. These three programmed necrosis pathways provide new targets in anticancer treatment, which may help to overcome the resistance of patients to anti-tumor therapy. Induction of programed necrosis via genetic or pharmacological approaches has been shown to provide anti-tumor potential in preclinical models of CRC. However, there is skepticism that drugs or treatments could also target cells other than tumor cells, such as immune cells and healthy intestinal epithelial cells. In addition, the pro-inflammatory effects of necroptosis, pyroptosis and ferroptosis should also be considered. Thus, it will be important in the future to design potent treatments that activate certain types of cell death, specifically in tumor cells, with rigorous safety testing.

## References

[1] Bischoff S.C., Barbara G., Buurman W., Ockhuizen T., Schulzke J.D., Serino M., Tilg H., Watson A., Wells J.M. (2014). Intestinal permeability—A new target for disease prevention and therapy. BMC Gastroenterol..

[2] Bray F., Ferlay J., Soerjomataram I., Siegel R.L., Torre L.A., Jemal A. (2018). Global cancer statistics 2018: GLOBOCAN estimates of incidence and mortality worldwide for 36 cancers in 185 countries. CA Cancer J. Clin..

[3] Fearon E.R., Vogelstein B. (1990). A genetic model for colorectal tumorigenesis. Cell.

[4] Patankar J.V., Becker C. (2020). Cell death in the gut epithelium and implications for chronic inflammation. Nat. Rev. Gastroenterol. Hepatol..

[5] Tang R., Xu J., Zhang B., Liu J., Liang C., Hua J., Meng Q., Yu X., Shi S. (2020). Ferroptosis, necroptosis, and pyroptosis in anticancer immunity. J. Hematol. Oncol..

[6] Koren E., Fuchs Y. (2021). Modes of Regulated Cell Death in Cancer. Cancer Discov..

[7] Fulda S. (2009). Caspase-8 in cancer biology and therapy. Cancer Lett..

[8] Li C., Egloff A.M., Sen M., Grandis J.R., Johnson D.E. (2014). Caspase-8 mutations in head and neck cancer confer resistance to death receptor-mediated apoptosis and enhance migration, invasion, and tumor growth. Mol. Oncol..

[9] Fritsch M., Gunther S.D., Schwarzer R., Albert M.C., Schorn F., Werthenbach J.P., Schiffmann L.M., Stair N., Stocks H., Seeger J.M. (2019). Caspase-8 is the molecular switch for apoptosis, necroptosis and pyroptosis. Nature.

[10] Linkermann A., Brasen J.H., Darding M., Jin M.K., Sanz A.B., Heller J.O., De Zen F., Weinlich R., Ortiz A., Walczak H. (2013). Two independent pathways of regulated necrosis mediate ischemia-reperfusion injury. Proc. Natl. Acad. Sci. USA.

[11] Sun L., Wang H., Wang Z., He S., Chen S., Liao D., Wang L., Yan J., Liu W., Lei X. (2012). Mixed lineage kinase domain-like protein mediates necrosis signaling downstream of RIP3 kinase. Cell.

[12] Vanden Berghe T., Demon D., Bogaert P., Vandendriessche B., Goethals A., Depuydt B., Vuylsteke M., Roelandt R., Van Wonterghem E., Vandenbroecke J. (2014). Simultaneous targeting of IL-1 and IL-18 is required for protection against inflammatory and septic shock. Am. J. Respir. Crit. Care Med..

[13] He G.W., Gunther C., Thonn V., Yu Y.Q., Martini E., Buchen B., Neurath M.F., Sturzl M., Becker C. (2017). Regression of apoptosis-resistant colorectal tumors by induction of necroptosis in mice. J. Exp. Med..

[14] Tan G., Huang C., Chen J., Zhi F. (2020). HMGB1 released from GSDME-mediated pyroptotic epithelial cells participates in the tumorigenesis of colitis-associated colorectal cancer through the ERK1/2 pathway. J. Hematol. Oncol..

[15] Li Y., Chen W., Qi Y., Wang S., Li L., Li W., Xie T., Zhu H., Tang Z., Zhou M. (2020). H2 S-Scavenged and Activated Iron Oxide-Hydroxide Nanospindles for MRI-Guided Photothermal Therapy and Ferroptosis in Colon Cancer. Small.

[16] Del Re D.P., Amgalan D., Linkermann A., Liu Q., Kitsis R.N. (2019). Fundamental Mechanisms of Regulated Cell Death and Implications for Heart Disease. Physiol. Rev..

[17] Kim E.H., Wong S.W., Martinez J. (2019). Programmed Necrosis and Disease:We interrupt your regular programming to bring you necroinflammation. Cell Death Differ..

[18] Vercammen D., Beyaert R., Denecker G., Goossens V., Van Loo G., Declercq W., Grooten J., Fiers W., Vandenabeele P. (1998). Inhibition of caspases increases the sensitivity of L929 cells to necrosis mediated by tumor necrosis factor. J. Exp. Med..

[19] Tonnus W., Belavgeni A., Beuschlein F., Eisenhofer G., Fassnacht M., Kroiss M., Krone N.P., Reincke M., Bornstein S.R., Linkermann A. (2021). The role of regulated necrosis in endocrine diseases. Nat. Rev. Endocrinol..

[20] Gong Y., Fan Z., Luo G., Yang C., Huang Q., Fan K., Cheng H., Jin K., Ni Q., Yu X. (2019). The role of necroptosis in cancer biology and therapy. Mol. Cancer.

[21] Annibaldi A., Wicky John S., Vanden Berghe T., Swatek K.N., Ruan J., Liccardi G., Bianchi K., Elliott P.R., Choi S.M., Van Coillie S. (2018). Ubiquitin-Mediated Regulation of RIPK1 Kinase Activity Independent of IKK and MK2. Mol. Cell.

[22] Gunther C., He G.W., Kremer A.E., Murphy J.M., Petrie E.J., Amann K., Vandenabeele P., Linkermann A., Poremba C., Schleicher U. (2016). The pseudokinase MLKL mediates programmed hepatocellular necrosis independently of RIPK3 during hepatitis. J. Clin. Investig..

[23] Tamura M., Tanaka S., Fujii T., Aoki A., Komiyama H., Ezawa K., Sumiyama K., Sagai T., Shiroishi T. (2007). Members of a novel gene family, Gsdm, are expressed exclusively in the epithelium of the skin and gastrointestinal tract in a highly tissue-specific manner. Genomics.

[24] Shi J., Zhao Y., Wang K., Shi X., Wang Y., Huang H., Zhuang Y., Cai T., Wang F., Shao F. (2015). Cleavage of GSDMD by inflammatory caspases determines pyroptotic cell death. Nature.

[25] Liu X., Xia S., Zhang Z., Wu H., Lieberman J. (2021). Channelling inflammation: Gasdermins in physiology and disease. Nat. Rev. Drug. Discov..

[26] Kesavardhana S., Malireddi R.K.S., Kanneganti T.D. (2020). Caspases in Cell Death, Inflammation, and Pyroptosis. Annu. Rev. Immunol..

[27] Burgener S.S., Leborgne N.G.F., Snipas S.J., Salvesen G.S., Bird P.I., Benarafa C. (2019). Cathepsin G Inhibition by Serpinb1 and Serpinb6 Prevents Programmed Necrosis in Neutrophils and Monocytes and Reduces GSDMD-Driven Inflammation. Cell Rep..

[28] Karmakar M., Minns M., Greenberg E.N., Diaz-Aponte J., Pestonjamasp K., Johnson J.L., Rathkey J.K., Abbott D.W., Wang K., Shao F. (2020). N-GSDMD trafficking to neutrophil organelles facilitates IL-1beta release independently of plasma membrane pores and pyroptosis. Nat. Commun..

[29] Evavold C.L., Hafner-Bratkovic I., Devant P., D′Andrea J.M., Ngwa E.M., Borsic E., Doench J.G., LaFleur M.W., Sharpe A.H., Thiagarajah J.R. (2021). Control of gasdermin D oligomerization and pyroptosis by the Ragulator-Rag-mTORC1 pathway. Cell.

[30] Kayagaki N., Kornfeld O.S., Lee B.L., Stowe I.B., O′Rourke K., Li Q., Sandoval W., Yan D., Kang J., Xu M. (2021). NINJ1 mediates plasma membrane rupture during lytic cell death. Nature.

[31] Rogers C., Fernandes-Alnemri T., Mayes L., Alnemri D., Cingolani G., Alnemri E.S. (2017). Cleavage of DFNA5 by caspase-3 during apoptosis mediates progression to secondary necrotic/pyroptotic cell death. Nat. Commun..

[32] Zhou Z., He H., Wang K., Shi X., Wang Y., Su Y., Wang Y., Li D., Liu W., Zhang Y. (2020). Granzyme A from cytotoxic lymphocytes cleaves GSDMB to trigger pyroptosis in target cells. Science.

[33] Zhang J.Y., Zhou B., Sun R.Y., Ai Y.L., Cheng K., Li F.N., Wang B.R., Liu F.J., Jiang Z.H., Wang W.J. (2021). The metabolite alpha-KG induces GSDMC-dependent pyroptosis through death receptor 6-activated caspase-8. Cell. Res..

[34] Hou J., Zhao R., Xia W., Chang C.W., You Y., Hsu J.M., Nie L., Chen Y., Wang Y.C., Liu C. (2020). PD-L1-mediated gasdermin C expression switches apoptosis to pyroptosis in cancer cells and facilitates tumour necrosis. Nat. Cell. Biol..

[35] Wang Y., Gao W., Shi X., Ding J., Liu W., He H., Wang K., Shao F. (2017). Chemotherapy drugs induce pyroptosis through caspase-3 cleavage of a gasdermin. Nature.

[36] Zhang Z., Zhang Y., Xia S., Kong Q., Li S., Liu X., Junqueira C., Meza-Sosa K.F., Mok T.M.Y., Ansara J. (2020). Gasdermin E suppresses tumour growth by activating anti-tumour immunity. Nature.

[37] LaRock D.L., Johnson A.F., Wilde S., Sands J.S., Monteiro M.P., LaRock C.N. (2022). Group A Streptococcus induces GSDMA-dependent pyroptosis in keratinocytes. Nature.

[38] Conrad M., Pratt D.A. (2019). The chemical basis of ferroptosis. Nat. Chem. Biol..

[39] Yang W.S., SriRamaratnam R., Welsch M.E., Shimada K., Skouta R., Viswanathan V.S., Cheah J.H., Clemons P.A., Shamji A.F., Clish C.B. (2014). Regulation of ferroptotic cancer cell death by GPX4. Cell.

[40] Bersuker K., Hendricks J.M., Li Z., Magtanong L., Ford B., Tang P.H., Roberts M.A., Tong B., Maimone T.J., Zoncu R. (2019). The CoQ oxidoreductase FSP1 acts parallel to GPX4 to inhibit ferroptosis. Nature.

[41] Doll S., Freitas F.P., Shah R., Aldrovandi M., da Silva M.C., Ingold I., Goya Grocin A., Xavier da Silva T.N., Panzilius E., Scheel C.H. (2019). FSP1 is a glutathione-independent ferroptosis suppressor. Nature.

[42] Kraft V.A.N., Bezjian C.T., Pfeiffer S., Ringelstetter L., Muller C., Zandkarimi F., Merl-Pham J., Bao X., Anastasov N., Kossl J. (2020). GTP Cyclohydrolase 1/Tetrahydrobiopterin Counteract Ferroptosis through Lipid Remodeling. ACS Cent. Sci..

[43] Brown C.W., Amante J.J., Chhoy P., Elaimy A.L., Liu H., Zhu L.J., Baer C.E., Dixon S.J., Mercurio A.M. (2019). Prominin2 Drives Ferroptosis Resistance by Stimulating Iron Export. Dev. Cell..

[44] Chen X., Kang R., Kroemer G., Tang D. (2021). Broadening horizons: The role of ferroptosis in cancer. Nat. Rev. Clin. Oncol..

[45] Yang J., Antin P., Berx G., Blanpain C., Brabletz T., Bronner M., Campbell K., Cano A., Casanova J., Christofori G. (2020). Guidelines and definitions for research on epithelial-mesenchymal transition. Nat. Rev. Mol. Cell. Biol..

[46] Wu J., Minikes A.M., Gao M., Bian H., Li Y., Stockwell B.R., Chen Z.N., Jiang X. (2019). Intercellular interaction dictates cancer cell ferroptosis via NF2-YAP signalling. Nature.

[47] Liu Z.G., Jiao D. (2019). Necroptosis, tumor necrosis and tumorigenesis. Cell. Stress.

[48] Feng X., Song Q., Yu A., Tang H., Peng Z., Wang X. (2015). Receptor-interacting protein kinase 3 is a predictor of survival and plays a tumor suppressive role in colorectal cancer. Neoplasma.

[49] Moriwaki K., Bertin J., Gough P.J., Orlowski G.M., Chan F.K. (2015). Differential roles of RIPK1 and RIPK3 in TNF-induced necroptosis and chemotherapeutic agent-induced cell death. Cell Death Dis..

[50] Li X., Guo J., Ding A.P., Qi W.W., Zhang P.H., Lv J., Qiu W.S., Sun Z.Q. (2017). Association of Mixed Lineage Kinase Domain-Like Protein Expression with Prognosis in Patients with Colon Cancer. Technol. Cancer Res. Treat..

[51] Bozec D., Iuga A.C., Roda G., Dahan S., Yeretssian G. (2016). Critical function of the necroptosis adaptor RIPK3 in protecting from intestinal tumorigenesis. Oncotarget.

[52] Zhao Q., Guo J., Cheng X., Liao Y., Bi Y., Gong Y., Zhang X., Guo Y., Wang X., Yu W. (2021). RIPK3 Suppresses the Progression of Spontaneous Intestinal Tumorigenesis. Front. Oncol..

[53] Alvarez-Diaz S., Preaudet A., Samson A.L., Nguyen P.M., Fung K.Y., Garnham A.L., Alexander W.S., Strasser A., Ernst M., Putoczki T.L. (2021). Necroptosis is dispensable for the development of inflammation-associated or sporadic colon cancer in mice. Cell Death Differ..

[54] Song M., Chan A.T., Sun J. (2020). Influence of the Gut Microbiome, Diet, and Environment on Risk of Colorectal Cancer. Gastroenterology.

[55] Keum N., Giovannucci E. (2019). Global burden of colorectal cancer: Emerging trends, risk factors and prevention strategies. Nat. Rev. Gastroenterol. Hepatol..

[56] Martens S., Bridelance J., Roelandt R., Vandenabeele P., Takahashi N. (2021). MLKL in cancer: More than a necroptosis regulator. Cell Death Differ..

[57] He S., Wang X. (2018). RIP kinases as modulators of inflammation and immunity. Nat. Immunol..

[58] Yu J., Li S., Qi J., Chen Z., Wu Y., Guo J., Wang K., Sun X., Zheng J. (2019). Cleavage of GSDME by caspase-3 determines lobaplatin-induced pyroptosis in colon cancer cells. Cell Death Dis..

[59] Tsuchiya K., Nakajima S., Hosojima S., Thi Nguyen D., Hattori T., Manh Le T., Hori O., Mahib M.R., Yamaguchi Y., Miura M. (2019). Caspase-1 initiates apoptosis in the absence of gasdermin D. Nat. Commun..

[60] Miguchi M., Hinoi T., Shimomura M., Adachi T., Saito Y., Niitsu H., Kochi M., Sada H., Sotomaru Y., Ikenoue T. (2016). Gasdermin C Is Upregulated by Inactivation of Transforming Growth Factor beta Receptor Type II in the Presence of Mutated Apc, Promoting Colorectal Cancer Proliferation. PLoS ONE.

[61] Xu S., He Y., Lin L., Chen P., Chen M., Zhang S. (2021). The emerging role of ferroptosis in intestinal disease. Cell Death Dis..

[62] Yang J., Mo J., Dai J., Ye C., Cen W., Zheng X., Jiang L., Ye L. (2021). Cetuximab promotes RSL3-induced ferroptosis by suppressing the Nrf2/HO-1 signalling pathway in KRAS mutant colorectal cancer. Cell Death Dis..

[63] Wu Y., Yu C., Luo M., Cen C., Qiu J., Zhang S., Hu K. (2020). Ferroptosis in Cancer Treatment: Another Way to Rome. Front. Oncol..

[64] Guo J., Xu B., Han Q., Zhou H., Xia Y., Gong C., Dai X., Li Z., Wu G. (2018). Ferroptosis: A Novel Anti-tumor Action for Cisplatin. Cancer Res. Treat..

[65] Zhang X., Sui S., Wang L., Li H., Zhang L., Xu S., Zheng X. (2020). Inhibition of tumor propellant glutathione peroxidase 4 induces ferroptosis in cancer cells and enhances anticancer effect of cisplatin. J. Cell. Physiol..

[66] Sato M., Kusumi R., Hamashima S., Kobayashi S., Sasaki S., Komiyama Y., Izumikawa T., Conrad M., Bannai S., Sato H. (2018). The ferroptosis inducer erastin irreversibly inhibits system xc- and synergizes with cisplatin to increase cisplatin′s cytotoxicity in cancer cells. Sci. Rep..

[67] Serebriiskii I.G., Connelly C., Frampton G., Newberg J., Cooke M., Miller V., Ali S., Ross J.S., Handorf E., Arora S. (2019). Comprehensive characterization of RAS mutations in colon and rectal cancers in old and young patients. Nat. Commun..

[68] Chen P., Li X., Zhang R., Liu S., Xiang Y., Zhang M., Chen X., Pan T., Yan L., Feng J. (2020). Combinative treatment of beta-elemene and cetuximab is sensitive to KRAS mutant colorectal cancer cells by inducing ferroptosis and inhibiting epithelial-mesenchymal transformation. Theranostics.

[69] Yang W.H., Ding C.C., Sun T., Rupprecht G., Lin C.C., Hsu D., Chi J.T. (2019). The Hippo Pathway Effector TAZ Regulates Ferroptosis in Renal Cell Carcinoma. Cell. Rep..

[70] Pan D. (2010). The hippo signaling pathway in development and cancer. Dev. Cell..

[71] Liu W., Chakraborty B., Safi R., Kazmin D., Chang C.Y., McDonnell D.P. (2021). Dysregulated cholesterol homeostasis results in resistance to ferroptosis increasing tumorigenicity and metastasis in cancer. Nat. Commun..

[72] (2020). Ferroptosis Is Inhibited in Lymph, Promoting Metastasis of Melanoma. Cancer Discov..

[73] Tasdogan A., Ubellacker J.M., Morrison S.J. (2021). Redox Regulation in Cancer Cells during Metastasis. Cancer Discov..

[74] Riera Romo M. (2021). Cell death as part of innate immunity: Cause or consequence?. Immunology.

[75] Biswas S.K., Mantovani A. (2010). Macrophage plasticity and interaction with lymphocyte subsets: Cancer as a paradigm. Nat. Immunol..

[76] Yatim N., Jusforgues-Saklani H., Orozco S., Schulz O., Barreira da Silva R., Reis e Sousa C., Green D.R., Oberst A., Albert M.L. (2015). RIPK1 and NF-kappaB signaling in dying cells determines cross-priming of CD8(+) T cells. Science.

[77] Aaes T.L., Kaczmarek A., Delvaeye T., De Craene B., De Koker S., Heyndrickx L., Delrue I., Taminau J., Wiernicki B., De Groote P. (2016). Vaccination with Necroptotic Cancer Cells Induces Efficient Anti-tumor Immunity. Cell. Rep..

[78] Snyder A.G., Hubbard N.W., Messmer M.N., Kofman S.B., Hagan C.E., Orozco S.L., Chiang K., Daniels B.P., Baker D., Oberst A. (2019). Intratumoral activation of the necroptotic pathway components RIPK1 and RIPK3 potentiates antitumor immunity. Sci. Immunol..

[79] Liu Z.Y., Zheng M., Li Y.M., Fan X.Y., Wang J.C., Li Z.C., Yang H.J., Yu J.M., Cui J., Jiang J.L. (2019). RIP3 promotes colitis-associated colorectal cancer by controlling tumor cell proliferation and CXCL1-induced immune suppression. Theranostics.

[80] Moriwaki K., Balaji S., McQuade T., Malhotra N., Kang J., Chan F.K. (2014). The necroptosis adaptor RIPK3 promotes injury-induced cytokine expression and tissue repair. Immunity.

[81] Orning P., Lien E., Fitzgerald K.A. (2019). Gasdermins and their role in immunity and inflammation. J. Exp. Med..

[82] Sharma B.R., Kanneganti T.D. (2021). NLRP3 inflammasome in cancer and metabolic diseases. Nat. Immunol..

[83] Zaki M.H., Boyd K.L., Vogel P., Kastan M.B., Lamkanfi M., Kanneganti T.D. (2010). The NLRP3 inflammasome protects against loss of epithelial integrity and mortality during experimental colitis. Immunity.

[84] Sharma D., Malik A., Guy C.S., Karki R., Vogel P., Kanneganti T.D. (2018). Pyrin Inflammasome Regulates Tight Junction Integrity to Restrict Colitis and Tumorigenesis. Gastroenterology.

[85] Dupaul-Chicoine J., Arabzadeh A., Dagenais M., Douglas T., Champagne C., Morizot A., Rodrigue-Gervais I.G., Breton V., Colpitts S.L., Beauchemin N. (2015). The Nlrp3 Inflammasome Suppresses Colorectal Cancer Metastatic Growth in the Liver by Promoting Natural Killer Cell Tumoricidal Activity. Immunity.

[86] Ghiringhelli F., Apetoh L., Tesniere A., Aymeric L., Ma Y., Ortiz C., Vermaelen K., Panaretakis T., Mignot G., Ullrich E. (2009). Activation of the NLRP3 inflammasome in dendritic cells induces IL-1beta-dependent adaptive immunity against tumors. Nat. Med..

[87] Lin K.H., Chang L.S., Tian C.Y., Yeh Y.C., Chen Y.J., Chuang T.H., Liu S.J., Leng C.H. (2016). Carboxyl-terminal fusion of E7 into Flagellin shifts TLR5 activation to NLRC4/NAIP5 activation and induces TLR5-independent anti-tumor immunity. Sci. Rep..

[88] Garaude J., Kent A., van Rooijen N., Blander J.M. (2012). Simultaneous targeting of toll- and nod-like receptors induces effective tumor-specific immune responses. Sci. Transl. Med..

[89] Lee P.H., Yamamoto T.N., Gurusamy D., Sukumar M., Yu Z., Hu-Li J., Kawabe T., Gangaplara A., Kishton R.J., Henning A.N. (2019). Host conditioning with IL-1beta improves the antitumor function of adoptively transferred T cells. J. Exp. Med..

[90] Derangere V., Chevriaux A., Courtaut F., Bruchard M., Berger H., Chalmin F., Causse S.Z., Limagne E., Vegran F., Ladoire S. (2014). Liver X receptor beta activation induces pyroptosis of human and murine colon cancer cells. Cell Death Differ..

[91] Yamazaki T., Hannani D., Poirier-Colame V., Ladoire S., Locher C., Sistigu A., Prada N., Adjemian S., Catani J.P., Freudenberg M. (2014). Defective immunogenic cell death of HMGB1-deficient tumors: Compensatory therapy with TLR4 agonists. Cell Death Differ..

[92] Wang W., Green M., Choi J.E., Gijon M., Kennedy P.D., Johnson J.K., Liao P., Lang X., Kryczek I., Sell A. (2019). CD8(+) T cells regulate tumour ferroptosis during cancer immunotherapy. Nature.

[93] Schnurr K., Borchert A., Kuhn H. (1999). Inverse regulation of lipid-peroxidizing and hydroperoxyl lipid-reducing enzymes by interleukins 4 and 13. FASEB J..

[94] Jiang X., Stockwell B.R., Conrad M. (2021). Ferroptosis: Mechanisms, biology and role in disease. Nat. Rev. Mol. Cell. Biol..

[95] Li W., Feng G., Gauthier J.M., Lokshina I., Higashikubo R., Evans S., Liu X., Hassan A., Tanaka S., Cicka M. (2019). Ferroptotic cell death and TLR4/Trif signaling initiate neutrophil recruitment after heart transplantation. J. Clin. Investig..

[96] Allam R., Kumar S.V., Darisipudi M.N., Anders H.J. (2014). Extracellular histones in tissue injury and inflammation. J. Mol. Med..

[97] Benson A.B., Venook A.P., Al-Hawary M.M., Cederquist L., Chen Y.J., Ciombor K.K., Cohen S., Cooper H.S., Deming D., Engstrom P.F. (2018). NCCN Guidelines Insights: Colon Cancer, Version 2.2018. J. Natl. Compr. Canc. Netw..

[98] Brown J.M., Attardi L.D. (2005). The role of apoptosis in cancer development and treatment response. Nat. Rev. Cancer.

[99] Jebelli A., Baradaran B., Mosafer J., Baghbanzadeh A., Mokhtarzadeh A., Tayebi L. (2021). Recent developments in targeting genes and pathways by RNAi-based approaches in colorectal cancer. Med. Res. Rev..

[100] Nehs M.A., Lin C.I., Kozono D.E., Whang E.E., Cho N.L., Zhu K., Moalem J., Moore F.D., Ruan D.T. (2011). Necroptosis is a novel mechanism of radiation-induced cell death in anaplastic thyroid and adrenocortical cancers. Surgery.

[101] Liu Z., Li Y., Zhu Y., Li N., Li W., Shang C., Song G., Li S., Cong J., Li T. (2022). Apoptin induces pyroptosis of colorectal cancer cells via the GSDME-dependent pathway. Int. J. Biol. Sci..

[102] Sui X., Zhang R., Liu S., Duan T., Zhai L., Zhang M., Han X., Xiang Y., Huang X., Lin H. (2018). RSL3 Drives Ferroptosis through GPX4 Inactivation and ROS Production in Colorectal Cancer. Front. Pharmacol..

[103] Chen C., Du W., Jing W., Sun P., Shi C., Zhang S., Liu Y., Cui P., Li A., Zhang R. (2022). Leveraging tumor cell ferroptosis for colorectal cancer treatment via nanoelicitor-activated tumoricidal immunity. Chem. Eng. J..

[104] McLane L.M., Abdel-Hakeem M.S., Wherry E.J. (2019). CD8 T Cell Exhaustion during Chronic Viral Infection and Cancer. Ann. Rev. Immunol..

[105] Yu J., Zhong B., Xiao Q., Du L., Hou Y., Sun H.S., Lu J.J., Chen X. (2020). Induction of programmed necrosis: A novel anti-cancer strategy for natural compounds. Pharmacol. Ther..

